# XML schemas and mark-up practices of taxonomic literature

**DOI:** 10.3897/zookeys.150.2213

**Published:** 2011-11-28

**Authors:** Lyubomir Penev, Christopher HC Lyal, Anna Weitzman, David R. Morse, David King, Guido Sautter, Teodor Georgiev, Robert A. Morris, Terry Catapano, Donat Agosti

**Affiliations:** 1 Bulgarian Academy of Sciences & Pensoft Publishers, Sofia, Bulgaria; 2 The Natural History Museum, Cromwell Road, London, UK; 3 Smithsonian Institution, Washington, DC, USA; 4 The Open University, Milton Keynes, UK; 5 IPD Böhm, Karlsruhe Institute of Technology, Germany & Plazi, Bern, Switzerland; 6 Pensoft Publishers, Sofia, Bulgaria; 7 University of Massachusetts, & Harvard University Herbaria, Boston, USA; 8 Plazi, Bern, Switzerland

**Keywords:** mark-up, XML schema, taxonomy, TaxonX, TaxPub, taXMLit

## Abstract

We review the three most widely used XML schemas used to mark-up taxonomic texts, TaxonX, TaxPub and taXMLit. These are described from the viewpoint of their development history, current status, implementation, and use cases. The concept of “taxon treatment” from the viewpoint of taxonomy mark-up into XML is discussed. TaxonX and taXMLit are primarily designed for legacy literature, the former being more lightweight and with a focus on recovery of taxon treatments, the latter providing a much more detailed set of tags to facilitate data extraction and analysis. TaxPub is an extension of the National Library of Medicine Document Type Definition (NLM DTD) for taxonomy focussed on layout and recovery and, as such, is best suited for mark-up of new publications and their archiving in PubMedCentral. All three schemas have their advantages and shortcomings and can be used for different purposes.

## Introduction

Traditional taxonomic publication has led to a vast quantity of valuable data effectively trapped in paper publications. Recent developments in transferring these to digital media, particularly using PDF format and placing them on the web, have increased overall access to publications dramatically but not taken taxonomic publication to a format appropriate to today’s methodologies of accessing and re-purposing data. Although simple searches of single or multiple documents may lead to the user finding the search terms in context, this context may not be what the user sought or, if the search is successful, the information sought (e.g. taxon treatments, specimen data) are not retrieved in a format suitable for repurposing (such as analysis of specimen data). To allow more precise searching for prioritised components of publications and retrieval of data in a format that is repurposable, taxonomic papers are being marked-up in XML and interfaces for queries being developed (Kirkup et al. 2005; Curry and Connor 2007, 2008; Agosti et al. 2007; Lyal and Weitzman 2008; Penev et al. 2010a; Willis et al. 2010).

The XML format has been identified as an important means of extending access to data from scientific papers (Murray-Rust and Rzepa 2002; Cui 2008b). Standards in XML for a range of taxonomic data have been developed through Biodiversity Information Standards (TDWG) such as for taxonomic names (Taxonomic Concept Transfer Schema), specimen data (ABCD, Darwin Core) and taxonomic descriptions (Structured Descriptive Data, SDD) (Hagedorn et al. 2005), for example, although so far there is no agreed-upon standard for taxonomic literature. An alternative to XML may be RDF, but there is less work done on RDF in the context of taxonomic literature; the relative merits and demerits of each will not be explored here, although it is worth noting that XML can be used as a stage in conversion to RDF where desired and appropriate (Cui 2008a; Cui et al. 2010a, and see below). XML mark-ups are currently being used both for new papers which are ‘born-digital’ and legacy literature, whose very varied structure poses much greater problems.

There are currently several different XML schemas and Document Type Definitions (DTD) (in the text, schema refers to both, unless specifically mentioned) being used for the mark-up of taxonomic literature, of which the three most widely used ones are discussed in this publication. The different schema designs reflect different priorities and consequently criteria for development. One distinction is whether the focus of the mark-up is on structure of the document as a whole (document-centric) or some part of the content of the document (content-centric). Another is the extent to which the marked-up text is potentially interoperable with (or using common elements with) other implementations. Notably, even with these distinctions, there are developing convergences between different approaches. An example of the content-centric approach is a focus on morphological descriptions (Heidorn et al. 2002; Cui and Heidorn 2007; Cui 2008a, b). In their work the publication is viewed more as metadata and the emphasis placed on the detail of morphological terms and the potential or repurposing the content. In this, the mark-up approaches SDD (Hagedorn et al. 2005), a schema produced explicitly for descriptive data. At the other extreme, some projects have employed a very generic schema to contain the document and structural information (i.e., pages, paragraphs, lines, headings, etc.) and used particular elements of taxonomic texts to assist in mark-up, relying on repeatable structural components of taxonomic descriptions (for example distributions, taxon names, morphological descriptions, stratigraphic detail, etc.) (Kirkup et al. 2005; Curry and Connor 2007, 2008). Weitzman and Lyal (2004) used a version of the TEI-Lite schema (http://www.tei-c.org/Guidelines/Customization/) with some taxonomy tags as an interim mark-up standard in the INOTAXA project. This is a very generic solution to properly model the complexity of taxonomic texts and, while the broader TEI tag set can certainly be customized for retrospective conversion of legacy taxonomic literature, TEI-Lite *per se* is not an ideal fit; the version of TEI-Lite created has not been used outside the INOTAXA project.

More elaborate schemas have been designed to have a wide application to legacy taxonomic literature, provide access to more detail, and incorporate bibliographic information about the publication that is at least compatible with standards used in other sectors (particularly libraries). TaxonX (http://www.taxonx.org, http://sourceforge.net/projects/taxonx) was created by an interdisciplinary group around Plazi (http://www.plazi.org, see also http://en.wikipedia.org/wiki/Plazi) (Agosti et al. 2007; Agosti and Egloff 2009). The goal of TaxonX is to model taxon treatments in publications to provide a basis for data mining and extraction, while generic textual features are given marginal importance. A further schema,taXMLit, (Weitzman and Lyal 2004) (http://www.sil.si.edu/digitalcollections/bca/documentation/taxmlitv1-3intro.pdf; http://wiki.tdwg.org/twiki/bin/viewfile/Literature/WebHome?rev=1;filename=taXMLit_v5-04.xsd) has been developed as part of the INOTAXA project (http://www.inotaxa.org). It was seen as a step towards developing an interoperable system allowing simultaneous access to both literature content and other data types such as specimen data and names. The goal is to provide very flexible possibilities for data mining though tagging a wide range of components within the taxonomic papers.

TaxonX and taXMLit are mark-up XML schemas developed primarily to encode historical (legacy) taxonomic literature (implying any text post-publication including modern texts, although neither has been used by publishers as a vehicle to deliver new publications). In contrast, the TaxPub DTD (http://sourceforge.net/projects/taxpub), an extension of the DTD of the US National Library of Medicine (NLM, http://dtd.nlm.nih.gov), has been developed specifically to facilitate mark-up of new, “born digital” taxonomic publications as part of the publication process. While TaxonX has been developed primarily to model treatments but model the entire publication at a very generic level, taXMLit and TaxPub provide an extensive tag set (in TaxPub’s case inherited from the base NLM DTD) for mark-up of generic (i.e., non Taxonomy-specific) document features, enabling location of relevant content throughout the document.

Once a document is marked-up into XML the full potential of that transformation can only be achieved through the creation of queries tailored to the schema elements. These can be incorporated into a portal for ease of human use, as well as built into web services. For TaxonX the portal is Plazi (http://www.plazi.org), for taXMLit the portal is INOTAXA (http://www.inotaxa.org).

An important aspect for use of a schema is the ease with which text may be parsed into it. A mark-up tool, GoldenGATE, was developed by Plazi (together with IPD Böhm at the Karlsruhe Institute of Technology, Germany) to facilitate this process (http://plazi.org/?q=GoldenGATE). Pensoft Publishers have developed the Pensoft Mark-up Tool (PMT) based on TaxPub for routine use in their publishing practices (Penev et al. 2010a; b). Cui (2008a) and Cui et al. (2010b) discussed a mark-up tool for species descriptions.

Sautter et al. (2007a) compared seven different schemas for mark-up of taxonomic publications: ABCD, SDD/UBIF, TaxonX, taXMLit, Linnaean Core, Darwin Core and NCD (Natural Collection Description). The authors concluded that only four of them – ABCD, TaxonX , taXMLit and SDD/UBIF, were appropriate for mark-up of taxonomic documents; the first three of them have been evaluated as more “document-centric” and the last one as clearly “data-centric”, the former being more optimal for mark-up of variously and inconsistently structured documents in the legacy literature than the latter. TaxonX and taXMLit have been analysed comparatively in order to investigate the possibility of mapping between them (Catapano and Weitzman 2007).

In this paper two schemas reviewed by Sautter et al. (2007a), ABCD (designed for specimen data) and SDD (designed for morphological descriptive data) are not considered further, as we assess them as much less appropriate for full mark-up of publications than the others. However, in the near future the relationships of the schemas designed for literature to more data-centric schemas, such as SDD and Darwin Core, should certainly be explored as being of primary interest for integration of “data-centric” and “document-centric” schemas.

The present paper aims at understanding the prioritized functions and scope of the three schemas most widely used for mark-up of taxonomic literature, namely TaxonX, taXMLit and TaxPub, and summarizes the experience and use cases accumulated during the four years following the analysis by Sautter et al. (2007a). In the context of an EU-funded project to support the development of virtual research communities involved in biodiversity science, ViBRANT, it is important to increase the compatibility of these schemas and this paper is a first step towards this.

## The concept of “taxon treatment”

Perhaps the most significant component of taxonomic literature is the ‘taxon treatment’: information about a single taxon, typically headed by the taxon name and including morphological, distributional, taxonomic and other information about that taxon. Taxonomic treatments are important because they permit labelling and delimiting a dedicated piece of information describing a taxon within a document from other similar pieces of information, describing other taxa. The retrieval of this content type has been identified as valuable to users of marked up text through formal and informal assessment (Parr and Lyal 2007), and the importance of enabling the user to retrieve a digitized taxon treatment as a core element has been recognised by most projects employing XML for taxonomic publications (e.g., Weitzman and Lyal 2004; Kirkup et al. 2005; Lyal and Weitzman 2008; Agosti et al. 2007; Sautter et al. 2007a). Subsequent usages of the marked-up paper, for example dissemination of content to various aggregators, can in some cases be performed at the level of treatments. In addition, marked-up text or data can be retrieved by machine from either within or outside treatments. Inevitably the concept of the taxon treatment is incorporated in most if not all schemas developed for taxonomic literature, both in the mark-up process and to inform user queries.

Determining the boundaries of taxon treatments in the mark-up process can be problematic and require manual intervention. Curry and Connor (2008) described the automatic identification and tagging of elements that typically occur within treatments, using stylistic rules to parse the text; they seem to have identified treatment boundaries *a priori*. More extensive algorithms also based on publication-specific stylistic rules (but not requiring *a priori* identification of treatment boundaries) were employed in a trial mark-up of a large single volume of the *Biologia Centrali-Americana* into taXMLit (Weitzman and Lyal 2006; Lyal and Weitzman 2008). The Plazi project atomises the publication into taxon treatments and, seek to maximize the number and consistency of tags by machine (either before or after publication) (Agosti et al. 2007; Catapano 2010; Penev et al. 2010a). The concept of taxon treatments from the viewpoint of their mark-up in taxonomic literature has been described by Catapano (2010) and Penev et al. (2010a). Therefore, we shall only briefly summarize the main features of treatments.

According to a definition by Norman Johnson (pers. commun.) adopted by Catapano (2010), a taxon treatment is a “publication or (more frequently) section of a publication documenting the features or distribution of a related group of organisms (called a “taxon”, plural “taxa”) in ways adhering to highly formalized conventions”. Some of these conventions (those pertaining to a subset of the treatment dealing with nomenclature) are maintained by scientific commissions accepted by the taxonomic profession, including the *International Code for Zoological Nomenclature* (ICZN) for animals, and the *International Code of Nomenclature for algae, fungi, and plants (ICNafp)*.

There is considerable structural diversity in taxon treatments across taxonomic literature, the main sources of variation being historical differences in the approach to treatments between different groups of taxonomists and across time, and different editorial and publishers’ formats. Nevertheless, it is possible to identify a few key features commonly found in treatments, such as the “Nomenclature” section, containing names and synonyms, “Material examined”, containing data on the studied specimens, “Type designation” (for new or revised taxa), “Morphological description”, “Etymology”, on the origin of the newly proposed names, “Differential diagnosis” separating the taxon from similar taxa, as well as data on biology, ecology, or conservation status, etc.

Penev et al. (2010a) listed the following cases in which a logically delimited block of text within a taxonomy paper can be regarded as a taxon treatment:

1. New taxon description or re-description of a known taxon

2. Change of a nomenclatorial status of a taxon (a nomenclatural act)

3. Summary of some or all previous knowledge on a taxon from literature sources, usually structured in logical pieces, e.g., nomenclature, morphological description, distribution, ecology, biology

4. Summary of some or all previous knowledge plus newly published data on the same taxon, e.g., localities, ecological/biological observations

5. Summary of newly published data on an already known taxon

6. Summary of treatments of subordinated taxa, for instance a revision or catalogue of a genus listing treatments of ALL or SOME of its species is a treatment of that genus

7. Listing of subordinated taxa, e.g., a checklist of a family from a region forms a treatment of that family.

At the same time, the following cases do not usually constitute a treatment:

1. A citation of a taxon name within a text, although such a citation usually holds information linked to the particular taxon. For instance, listing of a species within a “plain” checklist cannot usually be a treatment of that species (in early literature under the ICZN such an instance must be considered a treatment in certain circumstances); a sentence within a text paragraph stating that “taxon X is parasitic on taxon Y” is neither a treatment of taxon X nor of taxon Y.

2. An identification key, because in some cases keys are constructed for related taxa that do not form a taxon (they may form a “species-group” or “taxa-group”, but this is not a taxon unless a name is given to that group). Identification keys, even they are exhaustive for a named taxon, are usually tagged separately from taxon treatments. However, some keys include all of the information within a publication about a given taxon, and the practice may be to consider them treatments. In some cases keys include taxon treatments, including those of new taxa, or synonymies. How keys are tagged is probably an editorial matter.

3. A single picture or group of pictures of a taxon. In some early publications, however, a taxon is based exclusively on an image and its caption, a source which is available under the relevant Code, and therefore the picture and caption have to be regarded as a treatment.

4. A single map or group of maps of the occurrences of a taxon.

5. Gene sequence(s) of a taxon.

6. SDD (Structured Descriptive Data) (or any) matrices, or raw data, or databases. Treatments can be relatively easily generated from databases, however, and information on a taxon can be considered as becoming a treatment when (a) it is published, and (b) corresponds to the aforementioned description of a taxon treatment.

A publication may consist of one or many treatments of different taxa of different taxonomic ranks. One taxon may have more than one treatment within a publication, although the tradition of systematics publishing usually assumes one “core” treatment per taxon within a document. One treatment can include nested treatments, e.g., a genus and its species.

Implementation of the TaxonX schema and the TaxPub DTD largely follow the above restrictions. Implementation of taXMLit has been less restrictive in marking up complete papers, encompassing the less usual formats discussed above where appropriate, since more open-ended concepts of what makes a treatment have proven necessary, authors having been found to publish nomenclatural and taxonomic changes and treatments in a much wider variety of ways than listed in the more restricted list above. In the electronic era, broader notions of a treatment can easily be added to the electronic forms by simple extension of the schema or DTD.

## Descriptions of schemas

### 1. TaxonX

**1.1. Sources:**

http://sourceforge.net/projects/taxonx/; http://www.taxonx.org/schema/v1/taxonx1.xsd; www.plazi.org, Sautter et al. 2007a

**1.2. Description**

TaxonX is an XML schema for encoding taxonomic literature in order to:

• Create open, stable, persistent, full text digital surrogates of taxonomic treatments

• Identify taxonomic treatments and their major structural components to enable networked reference and citation

• Identify lower level textual data such as scientific names and localities (Darwin Core or any other relevant schema may be used), morphological characters, and bibliographic citations in order to facilitate their extraction by, and integration with, external applications and resources

• Study and describe the structure of systematics publications by creating few typical corpora of literature, such as entire journals (e.g., AMNH Novitates, Zootaxa), taxa (e.g., all ant systematics papers post 1995), or faunistic studies (e.g. all ant systematics paper covering Madagascar ranging from 1758 to 2011)

TaxonX is a lightweight (with only 30+ elements) and flexible schema for mark-up of treatments which can be quickly learned and may be applied to the wide variety of formatting present in legacy documents as well as new publications. In many cases it relies on use of external schemas for modelling certain kinds of information [e.g., the use of MODS (Metadata Object Description Schema: http://www.loc.gov/standards/mods/) for file level bibliographical metadata; Darwin Core for observation data: http://rs.tdwg.org/dwc/]. It has loose content requirements that allow for a wide variety of instances to be encoded over time and at many levels of granularity, while maintaining validity through iterations. Additionally, TaxonX contains mechanisms for semantic normalization of the data contained in treatments.

**1.3. Design and development**

Development of TaxonX began at the American Museum of Natural History (AMNH) and continued through the duration of a subsequent NSF/DFG grant (see below). As the project was concluding, participants established Plazi, a Switzerland-based independent not-for-profit organization aiming to help remove technological, social, and legal barriers to the creation of and access to taxonomic literature. Among its many activities, Plazi maintains the TaxonX schema and a repository of XML-encoded publications, develops the semi-automatic mark-up tool GoldenGATE (Sautter et al. 2007a), and strenuously advocates open access to scientific literature (Agosti and Egloff 2009).

TaxonX provides for the encoding of taxon treatments, with elements for the major structural components of treatments (e.g., Nomenclature, Materials examined, Description, etc.) and phrase-level features of interest in taxonomy (e.g., scientific names, locality names, characters, etc.) as well as mechanisms for linking to external resources and the semantic normalization of terms mentioned in the source document. The TaxonX instances encoded by Plazi contain a moderate degree of mark-up. Bibliographic metadata for the source documents are provided in each instance. Other sections of treatments are identified and named when they occur, but are not always present due to the wide variability of the structure of the source documents. All scientific names are marked and associated with an LSID, but other features may not always be identified. The section “Materials examined” can be broken down to individual materials citations, which in turn may normalized and linked to external resources, such as a type specimen, through LSIDs or other links.

A special emphasis has been given to link data to external resources, such as Life Science Identifiers (LSIDs). Tools in GoldenGATE have been developed to communicate automatically with external sources such as nameservers to retrieve LSIDs to taxonomic names in case they have already been entered, or to enter them upon discovery in an article, create the record and subsequently retrieve the LSIDs (e.g., in collaboration with the Hymenoptera Name Server), or on a manual base with Zoobank.

**1.4. Implementations**

**Use Case 1: The GoldenGATE (GG) software tool** (http://plazi.org/?q=Golden GATE). GG development is lead by Guido Sautter (Sautter et al. 2007b) to serve the mark-up of legacy literature. GG itself is highly flexible and integrates a set of tools and modules that allow highly automated large-scale output of documents marked in TaxonX or other XML schemas. The use cases listed below have been performed using GG. In 2010, GG launched a web interface to integrate social networking elements like crowdsourcing in the mark-up process.

**Use Case 2:** **Ants of Madagascar.** In 2006-2008, all available literature on the ants of Madagascar was OCR-ed, marked-up to the treatment level and stored on Plazi’s treatment repository; this comprises ca 4,000 treatments from ca. 2,500 pages extracted from 119 legacy publications with taxonomic descriptions.The project formed the basis for the subsequent development of Plazi’s mark-up projects (see below).

**Use Case 3:** **The Zootaxa-TaxonX-ZooBank Project.** In 2007, GBIF approved a Seed Money Award project entitled “Extracting Nomenclatural Data, Species Descriptions and Collecting Events from Legacy Publications: The Zootaxa-TaxonX-ZooBank Project” (GBIF Tracking Number 2007-94). Within this project, a TAPIR protocol has been developed for first time to render to GBIF occurrence data that have been marked up in taxonomic publications (http://data.gbif.org/datasets/provider/241).

**Use Case 4:** **SPM (Species Profile Model) export from Plazi to Encyclopedia of Life (EOL).** Plazi has developed a web service providing treatments in Species Profile Model (SPM) format allowing EOL and other interested parties, such as GBIF and others, to automatically harvest and consume content. Plazi received a small grant from EOL (managed by GBIF) to implement a service based on the SPM for the provision of taxonomic descriptions to EOL to complement a previous GBIF Seed Money Award to Zootaxa and Plazi that mobilised species occurrence records for the GBIF network (Use Case 3). The data for the project were taxonomic publications related to ants (Use Case 1). An XSLT conversion to SPM RDF/XML was developed and deployed as a web service using the eXist XML database (www.exist-db.org) so that SPM files generated dynamically from the TaxonX files can be retrieved via an HTTP GET request. A documented Application Programming Interface (API) is provided for the service, which allows the client applications latitude on tailoring the service. Sufficient documentation is provided so that clients can use the service for processing of the underlying XML document. At the date of writing (September 2011), 5892 treatments have been made accessible to EOL, including fish, ant and platygasteroid wasps.

**Use Case 5:** **Overall content in taxonX.** At the date of writing, 1,012 articles from 131 different journals and books spanning a period from 1758 to 2011 have been converted into TaxonX resulting in 15,863 treatments accessible on plazi.org. Most of the taxa covered are animals with an increasing number on plants and fungi taxa, (Plazi.org, accessed November 21, 2011).

**1.5. Problems encountered and lessons learned**

Based on accumulated experience, the following success factors of TaxonX can be summarized:

• It is a lightweight and flexible schema which can be quickly learned and may be applied to a wide variety of formatting found in legacy documents

• It relies on use of external schemata (see use of MODS for file-level bibliographical metadata).

• Its loose content requirements allow for instances to be encoded over time and at many levels of granularity, while maintaining validity through iterations.

• It contains mechanisms for semantic normalization of the data contained in treatments. See TaxonX‘s use of Darwin Core (soon perhaps Linnaean Core, SDD, etc.) to normalize phrase level data, and xid elements for inclusion of LSID‘s, ITIS, HNS, or other external identifiers.

However, there are also some hurdles for the adoption of TaxonX, such as:

• The heterogeneity and structural looseness of the data contained in some legacy taxonomic treatments makes encoding and semantic normalization even by a lightweight and flexible schema difficult and requires substantial expert intervention.

• The flexibility of the schema may present difficulties both in authoring and in profiling the encoded data for use by external applications as well as in conversion into other schemas/DTDs, but not at a very basic level, that is treatment and nomenclature element.

• Dependence on external schemas requires vigilance and active maintenance of the schema; may complicate long-term validation of instances; namespace wrangling makes authoring difficult

• Mark-up, even in a light way, needs some domain specific expertise, namely specific quality controls to assure that the elements are properly identified, and therefore costs time.

Potential users of TaxonX could be:

• Biodiversity Heritage Library would become much more useful if at least treatment boundaries, nomenclatural elements and respective names were to be marked-up and linked to the respective scan on BHL.

• Ultimately, one could envision this to be an intermediary step to extract and store the treatments in more powerful structures, such as databases. All the treatments are primarily linked to genetic, distributional, nomenclatural and other data via the taxonomic name applied to the treatment. At Antbase/HNS, this link is in a simple form already implemented by a link from each citation to the respective PDF copy of the referring page.

• Future aggregators of treatments might be institutions like ZooBank, or essentially dedicated databases allowing specific applications, like iSpecies (http://www.ispecies.org), or the Taxon Pensoft Profile (http://ptp.pensoft.eu), to collect the treatments and use them for specific purposes.

• All aggregators that will benefit from improved search, information retrieval, and information extraction.

### 2. TaxPub

**2.1. Sources:**

http://sourceforge.net/projects/taxpub/; Catapano 2010

**2.2. Description**

TaxPub was designed with the aim to enable the mark-up of new “born-digital” taxonomic literature that could forgo unnecessary variation in style and form and adhere to a limited set of data elements so as to lower costs of both authoring and processing. TaxPub is an extension of the Journal Publishing Tag Set of the U.S. National Library of Medicine’s Journal Archiving Tag Suite (see http://dtd.nlm.nih.gov/). For more details see Catapano (2010).

**2.3. Design and development**

Starting in 2008, TaxPub was designed and developed by members of Plazi with the assistance of experts from the U.S. National Center for Biotechnology Information. The TaxPub extension is maintained as an open source project at SourceForge (http://sourceforge.net/projects/taxpub/) inheriting from the base DTD an extensive and robust set of elements for generic textual structures while adding a small number of elements relevant to taxonomy. These include elements for mark-up of taxon names, citations to specimens and other material, and statements describing morphology, as well as for treatments and treatment sections. Further semantics may be applied to many elements through use of terms in external vocabularies (such as Darwin Core) as values of attributes (more details in Catapano 2010 and http://species-id.net/wiki/TaxPub).

TaxPub, being part of the National Library of Medicines Journal Article Tag Suite (JATS), has the additional advantage that it can directly be archived in PubMedCentral, one of the most secure existing archives and, as a consequence, its content is cross-linked with the huge body of biomedical literature stored therein.

**2.4. Implementations**

The first TaxPub encoded treatments were provided from the Ohio State University based “vSysLab” (Virtual Systematics Laboratory) presentation of data on wasps (Platygastroidea) described as part of the US National Science Foundation’s Planetary Biodiversity Inventories program (see http://vsyslab.osu.edu/home_page.html).

Soon after the initial release of TaxPub, Plazi was joined by Pensoft, the publisher of the online open access taxonomy journal ZooKeys, in a collaboration to integrate TaxPub into its publication workflow. The approach differed from OSU’s in applying mark-up to submitted manuscripts. Pensoft faced a set of challenges similar to those in retrospective conversion. Among them was the identification and encoding of treatments, scientific names, and bibliographic references. Developing their own software tool (Pensoft Mark-up Tool, PMT, see Penev et al. 2010a), in 2010 ZooKeys began to publish TaxPub versions of their articles. Although lacking a very fine-grained level of mark-up granularity (for example, <material-citation> is not used), the ZooKeys articles accomplish many of the goals of the TaxPub extension. Treatments are identified, and thus are directly and easily machine addressable, as are treatment sub-sections. All scientific names and name parts are tagged with <tp:taxon-name> elements. <tp:nomenclature-citation> elements include <tp:taxon-name> and link to full bibliographic entries, themselves marked up with <mixed-citation>. Specifically, because TaxPub motivated and enabled its use of the NLM DTD, ZooKeys and PhytoKeys articles are approved for display and archiving in PubMedCentral.

The ZooKeys’ exemplar papers (Stoev et al. 2010; Blagoderov et al. 2010b; Brake and von Tschirnhaus 2010; Taekul et al. 2010) are entirely based on revision #123 available from the SVN trunk of TaxPub (http://sourceforge.net/projects/taxpub). In fact, the present exemplar papers are the first published TaxPub articles in biodiversity science, intended to demonstrate the advantages of the XML-based mark-up and editorial workflow in the way biodiversity information is being published and disseminated.

**Use Case 1: Editorial use at Pensoft.** TaxPub is used to mark-up taxonomic papers during the editorial process, using the Pensoft Mark-up Tool (PMT). As a result, through PMT and InDesign, 3 electronic versions of a paper are generated and routinely published: (1) PDF identical to the printed version; (2) HTML to provide links to external resources and semantic enhancements to published texts for interactive reading; (3) XML version compatible to PubMedCentral archiving NLM DTD TaxPub extension), thus providing a machine-readable copy to facilitate future data mining.

Currently TaxPub is used routinely in the editorial process of six journals published by Pensoft:

• ZooKeys – www.pensoft.net/journals/zookeys

• PhytoKeys – www.pensoft.net/journals/phytokeys

• MycoKeys – www.pensoft.net/journals/mycokeys

• International Journal for Hymenoptera Research – www.pensoft.net/journals/jhr

• International Journal of Myriapodology – www.pensoft.net/journals/ijm

• Comparative Cytogenetics – www.pensoft.net/journals/compcytogen

In addition, the TaxPub DTD and some of its phrase-level elements, such as taxon names, are used in Pensoft’s ecology journals:

• BioRisk – www.pensoft.net/journals/biorisk

• NeoBiota – www.pensoft.net/journals/neobiota

• Nature Conservation  – http://www.pensoft.net/journals/natureconservation

**Use Case 2: Export of new taxa to EOL.** All new species descriptions in Pensoft journals are exported to EOL on the day of publication through a tool that maps the content to EOL elements; the file contains bibliographic metadata, taxonomic classification, species description and links to the species images. The exported XML file is harvested by EOL on a daily basis.

**Use Case 3: Export of taxon treatments to Plazi.** All taxon treatments identified within the XML file of a published paper are harvested by Plazi and uploaded to the Plazi Treatment Repository. Thereafter, treatments are available for use by various organizations and individuals, e.g., EOL.

**Use Case 4: Export of taxon treatments to the Wiki environment Species-ID** (http://species-id.net/wiki/Main_Page). All taxon treatments at the level of genera and species identified within the XML file of a published paper are exported to Species-ID through a special software tool, including images, keys and bibliographies. The citation template of the taxon’s wiki page automatically includes the original source (article) to provide a permanent scientific record, as well as all consequent contributors to the respective wiki page (Penev et al. 2011).

**Use Case 5: Archiving in PubMedCentral.** ZooKeys was accepted for indexing and archiving in PubMedCentral in August 2010, followed by PhytoKeys. Since then TaxPub XML output of ZooKeys issues 50-54 has passed 4 rounds of testing at NLM. All suggestions have been implemented in the XML export and, where needed, corrections implemented in TaxPub.

**Use Case 6: Use of TaxPub XML files to create a semantically enhanced HTML version of the publication.** The process was described and exemplified in issue 50 of ZooKeys (Penev et al. 2010a, b); from then it has become routine practice for several of Pensoft’s journals (list provided above in the text).

**Use Case 7: Acceptance of manuscript in XML by Journal.** InZooKeys 50, Penev et al. (2010b) and Blagoderov et al. (2010a) piloted acceptance of manuscripts in XML format, generated from two independent sources: Scratchpads (sample papers: Blagoderov et al. 2010b; Brake and Tschirnhaus 2010) and the SysLab tool from the Hymenoptera Online database (Taekul et al. 2010). This process should become routine practice during the ViBRANT project.

### 3. taXMLit

**3.1. Sources:**

http://wiki.tdwg.org/twiki/bin/viewfile/Literature/WebHome?rev=1;filename=ta XMLit_v5-04.xsd; Weitzman and Lyal (2004)

**3.2. Description**

The taXMLit schema is designed to accommodate taxonomic literature. It was developed particularly in the context of Zoological and Botanical publications and should also be applicable to publications on fungi and palaeontology, although this has yet to be tested. The schema does not take into account the kinds of data needed for viral or bacterial publications. It covers all of the components of taxonomic publications and the taxon treatments contained within them, but does not encode individual character statements, which are dealt with by other projects such as SDD.

The schema is highly atomised, permitting both recovery of publication components (e.g. taxon treatments, diagnostic keys, images, bibliographic entries, discussion paragraphs) and of data within those components, such as specimen data, biological associations, atomised taxonomic names, and nomenclatural and taxonomic acts. It can be applied to the entire text of a publication and not only formal treatments as discussed above. The richness permits full application to any legacy format so far encountered. The full taXMLit contains data elements extracted from the text that permit detailed data querying, browsing, and download; a version that does not include the respective elements and is more document-centric has also been developed (‘taXMLite’: http://wiki.tdwg.org/twiki/bin/viewfile/Literature/WebHome?rev=1;filename=taXMLite_v5-04.xsd). This was developed to permit preliminary mark-up and subsequent upload access through the INOTAXA interface developed for taXMLit (see below); it is not discussed further here.

Implementation of the schema in an appropriate system (‘INOTAXA’ – http://www.inotaxa.org has been designed for this purpose) allows the text of marked-up taxonomic publications to be fully humanly searchable. In INOTAXA users may chose to view and download data (e.g. taxonomic names, specimen data, citations, biological association data, persons’ names) for use in analysis or other applications, or access taxon treatments, keys, images, or other content components as reference resources. In conjunction with the appropriate system, the schema would also facilitate static links from the text to other data sources (e.g. specimen databases on the web, ZooBank). Use of the schema for multiple taxonomic works allows these to be searched or browsed simultaneously, and permits links between different works that cover the same taxa or their synonyms. Moreover, this paves the way for uses to create virtual compilations of taxon treatments, comprising components of more than one original work, e.g. checklists, faunas, and floras. These applications require that the schema should, in the appropriate parts, use elements the same as or similar to those in schemas used by other relevant systems, and be mappable to them.

**3.3. Design and development**

TaXMLit and INOTAXA were conceived in 2001 in a Mellon-funded meeting focussing on the potential for combining information, literature, and research data, and funded in 2001 by the Atherton Seidell Fund. The project initially selected the *Biologia Centrali-Americana* to use in trials (57 volumes, more than 50,000 taxon treatments) with a wide coverage of animals and plants and a variety of editorial styles applied. This provided a varied base for testing the schema and also developing a called-for resource. Subsequently a number of other texts published between 1758 to 2008 and including formal taxonomic publications, catalogues and other formats have also been marked up to provide an even stronger test of applicability of the schema. Some of these are currently accessible through the INOTAXA.org pilot (currently accessible are two papers from Zootaxa, the more recent being Pyle et al. (2008) on *Chromis*, a paper that has been used by a number of initiatives to enable comparison); others will be made available in the near future.

An initial problem was source quality. Most legacy literature is not born digital, and incorporates outdated fonts, complex terms not easily resolvable by OCR, diacritic marks and other problematic aspects. Tests against BHL content in 2009 indicated a success rate for correct recognition of the scientific name of only 14-35% (Weitzman, unpublished; Freeland, unpublished). Morse et al. (2009) examined this problem and presented some solutions to be incorporated in the ViBRANT workflow. To date, mark-up to taXMLit has been undertaken through preliminary mark-up to TEI-Lite with a systematic ‘flavour’ (created by Weitzman and Lyal), and subsequent parsing to taXMLit, this being been either manual or automated through use of a purpose-written script based on stylistic features and landmarks introduced in the TEI-Lite mark-up.

Within taXMLit each text paragraph in the original publication (i.e., any text component terminated by the stroke of an ‘Enter’ key) is captured entire and given an ElementID, which run sequentially through the text. This facilitates later reconstruction of the order of the text components. In some cases, reconstruction will require a different order than the original. For example, polytomous keys, which have the structure of a tree, can be spread throughout the text with contrasting statements at the same level (called ‘lugs’ by taxonomists) separated by treatments or other complex elements, but need to be reconstructed without these interruptions. Individual paragraphs are then be parsed into more or less detailed elements as required. The ElementID allows the use of an IDREF attribute (a cross-reference within the mark-up). The full set of elements within taXMLit is large, designed to accommodate the atomisation of many elements (taxonomic names, for example, are fully atomised, with a rank assigned to each component) and provide the detail required for search, browse and download of identified components. While taXMLit uses elements that cover the same concepts as those used in other schemas (e.g. ABCD and Darwin Core, designed for specimen data), the individual elements are not all exactly the same, because the data as presented in the literature may be different in format from those recovered from specimen labels, for example, and may not be as easy to interpret. However, taXMLit is designed to permit mapping to ABCD and Darwin Core.

Much taxonomic literature employs abbreviations as standard (e.g. for genus names after the first use, or author names) and descriptors may be omitted (e.g. for suprageneric hierarchical ranks, or for repeated components of label data). While this information is simple to interpret for a human reader, it is less accessible to machine processing or amenable to database storage. For this reason taXMLit uses the attribute ‘Explicit’ with many elements to denote whether the information included is explicitly stated or implicit and derived either by programming code or by a human in the final mark-up verification.

The use of ‘Implicit’ is intended as a matter of project policy to accommodate only unequivocal interpretation (e.g. the abbreviation “A.” in front of a species name where the only genus name in context is “A-us” marked as that name). While editorial practice is to limit interpretations of the text drawing on information and knowledge from outside the text itself, the schema includes an element to accommodate alternative spellings of the same name included in a single text, to capture interpreted place names and, for added geographical coordinates, using a ‘source’ attribute. The facility for retrieving such interpretations is being developed.

Some of the original formatting is retained (e.g. underlining, italics, bold etc), although font and line indentation, for example, are not. Page numbers are retained.

As described by Parr and Lyal (2007) a formal assessment of user needs including taxonomists and some other groups was carried out as part of the development, and part of the testing of each phase of the INOTAXA build was carried out by taxonomists and others new to the system. The elements of taXMLit, the selection of elements to index in the INOTAXA database, and the query and browse functionalities of the INOTAXA interface, were designed in concert with this user assessment.

To support querying and browsing content, search speed is maximised by storing the marked-up texts in a relational database. Fifty-seven of the fields are indexed to permits Boolean searches. To date, upload to the database has been via individual scripts, but the database has recently been simplified and made scalable, and a generic upload tool is being built.

**3.4. Implementations**

**Use Case 1:** **Mark-up of ‘old’ taxonomic literature.** Literature used for this is primarily the *Biologia Centrali-Americana* (BCA) (http://www.sil.si.edu/digitalcollections/bca), but also employed other papers including parts of Linnaeus 1758 *Systema Naturae* and Linnaeus 1752 *Species Plantarum*. The lessons learned enabled the schema to be developed to deliver the flexibility required for older literature written before more modern standardization and the advent of the nomenclatural codes.

**Use case 2: Mark-up of recent taxonomic zoological and botanical taxonomic literature**. Fourteen texts in different formats were marked up spanning the dates 1992-2008, including ‘standard’ taxonomic papers from the Coleopterist’s Bulletin, Mosquito Systematics, Proceedings of the Biological Society of Washington, Systematic Botany, Transactions of the American Entomological Society and Zootaxa, part of a synonymic catalogue and a book chapter. As with older pre-Code texts, lessons learned enabled the schema to be refined to accommodate variation in modern literature, and manage multiple publications including treatments of the same taxa under the same and different names and in different systematic placements.

**Use case 3:** **Storage of mark-up.** To enable rapid search and retrieval of marked up content in a scalable manner a database with selected (high-usage) fields indexed was constructed in MySQL. This permits much more rapid access and retrieval of simple and complex queries than would be possible from storage as simple XML documents.

**Use case 4:** **Human search and browse of content.** The INOTAXA interface to content in taXMLit was built in several phases with testing of each phase primarily by taxonomists who were new to the system. The prototype includes three publications (a BCA volume on Coleoptera: Curculionidae, a Zootaxa paper on Curculionidae taxa also included in the BCA volume, and Pyle et al. (2008) on *Chromis* fish), together including more than 800 taxon treatments (Weitzman and Lyal 2006; Lyal and Weitzman 2008). Two additional sources of information were added: the digitised contents of Vaurie and Selander (1971) (georeferenced localities for specimens in the BCA) and a list of person names in all possible formats (e.g. Smith, Smith, J., J. Smith, J Smith etc) – this allows expressing synonymy of different name strings representing the same individual without editing / changing the original text. A link between the treatment retrieved and the treatment in the original text in PDF or JPEG format is available through the interface, as are links to any original images. Further information on INOTAXA and the queries that it permits is available at http://www.inotaxa.org.

**Use case 5: Availability of content to Encyclopedia of Life.** Currently marked-up text is mapped to the EoL schema and delivered to EoL with associated images for display on their pages (866 pages). The process is automatic and will deliver further pages on the next data upload to INOTAXA.

**3.5. Problems encountered and lessons learned**

• Interoperability. So that the schema could potentially deliver data in a format usable by other applications two choices were available: to incorporate elements of the target schemas or develop new schema elements within taXMLit that could be mapped to others. The latter was selected with the logic that taXMLit could be versioned as a stand-alone entity and updated by users as appropriate, without having to accommodate independent changes by embedded schemas.

• GUIDs. Initially unique identifiers were not explicitly included; however, as biodiversity informatics has moved towards implementation, a placeholder for GUIDs has been included in many elements.

• Accommodating multiple formats of legacy literature. Although taxonomic literature is reputedly standardized in content, experience with many different papers and books has demonstrated the extreme variability of formatting and structure applied, even within single papers. To accommodate the observed variation most of the complex elements of taXMLit are optional and available in many different places within the schema.

• Implicit content. Much content is implicit in nature (see discussion above). Care must be taken in recognizing such content, but it is necessary to do so to facilitate searching and browsing functionality in the interface, and even to retrieve some taxon treatments. Such implicit content is indicated as such in display by the use of a different font colour and annotation.

• Policy on correction of errors. Because spelling errors and other infelicities in the original publication may have nomenclatural significance, and because correction relies on individual expertise, apparent errors are not changed in the current implementation of taXMLit and INOTAXA. Such change or annotation must be explicitly authored, and the ability to do this will be introduced in a later implementation.

• Mark-up. Semi-automated mark-up has been achieved using a purpose-written script, incorporating rules developed to accommodate the structure of the individual publication. Even with this, there are places where specialist knowledge is required. To facilitate this, a SpecialistReview attribute has been introduced throughout the schema.

• Recovery of original formatting. Only some of the original formatting is retained, where this aids in understanding (e.g. italicisation). INOTAXA delivers content in a standardised format to aid comprehension, but allows (subject to copyright) access to the original text.

• Hierarchies. Each publication marked up in taXMLit inevitably has an independent taxonomic hierarchy, which is displayed in INOTAXA. Where a work is produced in multiple fascicles, it is assumed unless stated otherwise that the hierarchy does not change.

### Evaluation, comparison and cross-points between taxonX, TaxPub and taXMLit

The three schemas discussed above serve different purposes, but to an extent have to address the same issues. One is the identity of the communities who will use the output, and an understanding of the uses to which this output will be put. Further user needs analysis would be valuable, including building on Parr and Lyal’s (2007) analysis. So far, there has been no published study that explicitly makes use of marked up literature (although the number of views of content harvested by EOL from INOTAXA, Plazi and Pensoft indicate that this product at least is valued).

One question arising from a consdideration of meeting user needs relates to the size of the data ‘packages’ identified by elements within the schemas, a ‘package’ being a logical unit of information delivery enabling reuse. Packages discussed above include the taxon treatment, collection data for a single specimen, taxon name and publication citation, among others. The schemas discussed target different sizes of packages, taXMLit opting for the largest number and smallest packages, although these are nested within larger more encompassing packages (e.g. taxon treatments). Interoperability with non-literature schemas seems to require a high degree of atomisation (Lyal and Weitzman 2008). A related issue is that while data may be extracted from a publication (such as locality data for a specimen) the relevant metadata that are given elsewhere in the text (such as confidence limits in a georeference) may not be associated.

The complexity and atomisation of the mark-up (number, size and nesting of data packages) is likely to be proportional to the cost of mark-up, which will differ between the three schemas. A cost-benefit analysis may be helpful, although would need to be in the context of the uses planned for the marked up text (see below). There are prospects to reduce costs through automation of the different phases (Curry and Connor 2007; Sautter et al. 2007b; Morse et al. 2009; Cui et al. 2010a).

**Figure 1.**
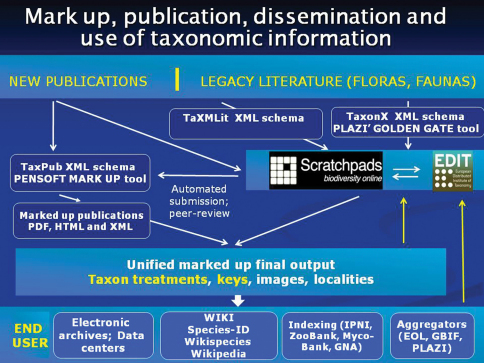
Flowchart of mark-up, publication, dissemination and use of taxonomic information. Scratchpads (http://scratchpads.eu/) and EDIT Cybertaxonomy Platform (http://wp5.e-taxonomy.eu/) stand for the community-based collaborative platforms for taxonomists developed by the EDIT FP6 project (http://www.e-taxonomy.eu/).

One of the strategic goals of biodiversity informatics is an increase in accessibility, compatibility and interoperability of data originating from different sources. If elements of taxonomic information coming from different sources are compatible (and thus can be made interoperable) they can then be easily harvested, indexed, collated, used and reused. The format of the final output of the individual schemas – in a form of XSLT stylesheets for instance – will be determined by the expectations and needs of the end users (Fig. 1).

In the context of schemas for taxonomy mark-up, compatibility is understood here as the *ability of the schemas to identify, mark-up and export elements used in both legacy and prospective taxonomic literature and needed for data mining and reuse by users*. An important criterion of compatibility is that schemas can be mapped to a shared (TDWG) vocabulary, thus allowing conversion between both literature schemas and others. Table 1 presents a rough evaluation of the schemas under consideration here with regard to a set of criteria that might prevent or facilitate generating a unified output from different taxonomic sources (and marked up with different schemas).

TaxonX and TaxPub are largely interoperable since both have been developed by the same author and contributors, and also because both schemas have been used together in some of the cases mentioned above. The challenge will be to ensure output compatibility between taXMLit and the others, particularly with TaxonX.

The schemas themselves are only part of the necessary comparison with respect to mark-up. Given the various complexities and challenges faced in the process of retrospective mark-up, different teams are developing different protocols and editorial decisions. Some of these have been indicated above. Table 2 provides comparison of some of the critical decision areas.

## Conclusions

There is a rich legacy of several hundred years of taxonomic literature. In addition to this, many new papers are published each year, driven by the recording of an estimated ca. 17,000 new taxa being described with some unknown number of re-descriptions. We can use technology to help us process this data overload, but only if we can first impose some form of structure on the data that facilitates machine-processing. Applying structure to a text is a remarkably challenging activity. This paper has considered three XML schemas devised to help address this problem.

Table 1 shows that the three currently most widely used schemas - TaxonX, TaxPub and taXMLit - cover the key text elements used by taxonomists fairly equally. This is encouraging as it suggests interoperability should be achievable among the schemas. Equally, it might lead one to ask why there should be three separate schemas.

The answer lies in Table 2. This table shows a greater range of answers to its questions, each schema with its own strengths, weaknesses and associated editorial practices. Reading this table in conjunction with the main text of this paper, we can see that each schema is focused on a different user need.

TaxonX addresses core data requirements of working taxonomists. Table 2 shows that it focuses on the three core elements required by taxonomists: treatments, names, and taxonomic and nomenclatural acts. Its use in Plazi has shown how it can successfully meet this basic user need. The focus on taxon treatments has led to some exciting developments towards making the data available as RDF triples in conjunction with GBIF or through the Species Model transfer (SPM) to the Encyclopedia of Life. This development will permit greater linking of taxonomic data across repositories.

TaxPub addresses the need to ensure that data in new publications is immediately accessible. Being specifically targeted at new literature, it can avoid many of the problems applicable only to historic literature (leading in Table 2 the frequent statement ‘n/a in prospective publishing’). This focus has allowed TaxPub not only to be successfully piloted as a publication tool, but for systems using the schema to automatically populate other resources, such as Plazi and Species-ID, and the prospect of generating treatments from and uploading to databases. Hence, data in the new text are immediately available for other researchers. In addition, table 2 shows it is the schema most suited to archival use as befits a schema targeted at publication and derived from JATS (Journal Archiving and Interchange Tag Suite - http://dtd.nlm.nih.gov/).

TaXMLit provides the richest mark-up of the three schemas. Table 2 shows it handles a greater range of data and in more detail than the other schemas and, for example, is the only schema that can handle a change in geographic place name since publication. However, this richness comes at a cost, since to efficiently exploit the data within a series of taXMLit texts they are ideally converted to a searchable database (as exemplified by INOTAXA), and to create a fully marked-up text is both time consuming and requires expert input. The future development goals for taXMLit include greater automation of mark-up, and a possible lightweight derivative taXMLite. The full taXMLit schema best serves the needs of a wide variety of researchers, and for those who wish to trawl the data as opposed to answer pre-defined questions, such as those working on the impact of climate change.

Therefore, we may conclude that having three solutions to the one problem of marking up taxonomic literature is appropriate because each schema addresses a different user need. TaxPub is the most suitable for born-digital literature, and the mark-up can be achieved at relatively low cost. There are few technical hurdles, for example, as the source material is already in digital format; and if there are ambiguities in the text they can be presented to the author(s) while they prepare their text. However, in focusing on new literature, TaxPub is not meant for handling historic texts (although there is an archival version in JATS that is designed for legacy literature, and might not only be used for mark-up but could also be submitted to PubMedCentral; this version, however, has not yet been customized for taxonomy). In contrast, both TaxonX and taXMLit can handle the issues that accompany historic texts. TaxonX focuses on taxon treatments, whereas taXMLit covers all data within a text. Hence, TaxonX is easier and cheaper to mark-up, but the results are not as widely usable as they would be had the original text been marked up in taXMLit. There is a clear need to understand the cost-benefit of marking up texts to assist users to decide which of the two schemas is more appropriate for them.

All three schemas have a role to play in ViBRANT. Both TaxonX and taXMLit could benefit from ViBRANT’s investigations into the use of citizen scientists to review texts and the use of automatic tools for data mining historic literature. This aims to enhance the accuracy of the data extracted, and to reduce the cost and time required to produce the mark-up. TaxPub at the same time will allow ViBRANT to publish its content in a semantically enhanced and state of the art way that not only provides the already proven option for easy dissemination of its content as well as provide a stable archive of the valuable content created through ViBRANT’s infrastructure.

This paper has discussed a means of achieving more use of the data in taxonomic literature by making that data easier to share, search, link, and combine, especially through semantic enhancement, and by exposing the data to new automated analytical techniques such as data mining. To achieve these goals, it is necessary to apply some form of structure to the literature. In the context of taxonomic literature mark-up we are fortunate to have seen the development of these three schemas to apply structure, for each addresses a particular user need. In addition, the schemas’ common coverage assures us that the core data they contain can be converted from one schema to another, and so could be equally accessible to any tool-sets developed to exploit each schema. This is true now of marked up taxonomic literature and is also true of future marked up taxonomic literature, whether newly written born-digital texts or digitised historic texts. These are the tools to support our advance towards liberating the data stored in taxonomic literature or to prevent their confinement from begin with.

**Table 1.** Evaluation of the three most widely used schemas for taxonomy mark-up (taxonX, TaxPub, taXMLit) with regard to availability of key text structure elements. Legend: “-” absent; “+” present, but needs further development; ++ available. Notes in table: ^1 ^TaxonXhas a focus on treatments;^ 2^ taXMLIt bibliographic metadata can be mapped to MODs etc; ^3^ taXMLit recognises a more inclusive definition of taxon treatments and marks all the same way; ^4^taXMLit marks citations, nomenclature, specimens, distributions and elements within these in detail, and identifies paragraph types; no further granularity is planned; ^5^The intention is that it can be mapped; ^6^Can be mapped to DC2; ^7^Reference lists, in-text citations of bibliographic references, but only generic link between both.

**Criteria**	**Taxon X**	**TaxPub**	**taXMLit**
Overall structure of document captured	n/a^1^	++	++
Bibliographic metadata: uses / can be mapped to current widely adopted standards (NLM, BibTex, MARC, MODS, etc)	++	++	+^2^
Taxon name mark-up fully granular, including names, ranks and authorities	++	+	++
Nomenclatural acts: use of controlled vocabularies and normalized (standardized) tags for different acts	+	+	+
Taxon treatments (as defined in text) delimited within texts	++	++	++^3^
Internal structure of treatments – level of mark-up granularity	++	++	++^4^
Nomenclature section of treatments (names, authorities, synonyms separately tagged)	++	++	++
Species occurrence data (Localities): compliance to Darwin Core; formats for use of geographical coordinates	++	++^5^	++^6^
Reference lists, in-text citations and links between both	+^7^	++	++
Accommodates persistent identifiers (UUID, GUID, LSIDs, DOI etc.) to identify different elements (taxon names, publications, treatments, datasets, keys, phylogenetic trees etc.)	++	++	+
Permits annotation so original text and annotation both visible to user	+/-	n/a	+

**Table 2.** Evaluation of editorial and mark-up practices employed by main users of the three most widely used schemas (taxonX, TaxPub, taXMLit) in retrospective mark-up of historical taxonomic literature. Legend: “-” weak; “+” present, but needs further development; “++” good; “+++” very good.

Criteria	taxonX	TaxPub	taXMLit
1) What are the tolerances for text accuracy?	Generally high – structural mark-up generation is mostly independent of text, detail mark-up to some degree depends on text accuracy - text accuracy is checked during mark-up generation	n/a in prosepective publishing.	Text accuracy managed through pre-mark-up checking manually and through processes developed in ABLE project (Morse et al. 2009)
2) What are the editorial policies for, among others:			
a) corrections/retention of typos and other errors in the text	Retained if in original publication	n/a in prospective publishing	Retained If in original publication; some corrections marked as implicit if unequivocal.
b) interpretation of unclear text	What is “unclear text”? Abbreviated or omitted taxonomic epithets are disambiguated or filled in, respectively, during mark-up generation	n/a in prospective publishing	Use ‘implicit’ attribute for unequivocal clarification; if reliant on subjective interpretation not changed.
c) choice of “copy-text”, i.e., the exemplar from which the digitized version of the text will be made. It is highly unlikely that every copy of any edition of a work will have exactly the same text	Probably not relevant to TaxonX – most documents marked are journals or journal articles, which extremely rarely have more than one edition	n/a in prospective publishing	Source copy text in large institutional libraries. Possibility for multiple copies of same work to be uploaded if marked up. Within texts treat cancels and cancellands separately.
3) What are the policies and practices for normalization and other annotation, such as:			
a) expansion of abbreviations	Abbreviations get tagged, data they imply stored in DwC children of dedicated tax:xmldata element, but not widely used as of yet	n/a in prospective publishing	Use of ‘implicit’ attribute for unequivocal expansions (e.g. generic names, author names)
b) normalization of taxon names, personal names, corporate names, etc.	Taxon names atomized, epithets expanded or filled in where abbreviated or missing, normalized epithets stored in DwC children of dedicated tax:xmldata elementNo normalization for person or corporate names as of yet	n/a in prospective publishing	Primarily reproduced as original; in some cases for both taxon names and person names, some normalization occurs in a separate part of the mark-up. Facility for linking synonyms of Parties / Agents outside text in place through INOTAXA.
c) modernization of archaic or changed place names (e.g., Rhodesia/Zimbabwe)	None as of yet	n/a in prospective publishing	Primarily reproduced as original; also has ability to capture an ‘interpreted’ place name. Facility for searching forms of changed place names being developed in INOTAXA.
d) annotation and other editorialization, as for example, correction of incorrect taxon names, assignment of coordinates to location names	Actual taxon name stays as in original publication, normalized epithets stored in DwC children of dedicated tax:xmldata element usually contain correct value	n/a in prospective publishing	Primarily reproduced as original; also has ability to capture corrections and additions as ‘interpreted’ data and, for added coordinates, using a ‘source’ attribute,
4) What are the textual objects of interest which will be encoded (i.e., do not aim to tag everything). What is in scope, and what is not? What has the highest priority?			
treatments	+++	+++	+++ (high)
keys	+	++	+++ (high)
Phylogenetic and other trees	+	+	++ (low)
Front and back matter	-	+++	+++ (medium)
Discussion paragraphs	++	+++	+++ (medium)
Names	+++	+++	+++ (high)
Specimen data	++	++	+++ (high)
Taxonomic and nomenclatural acts	+++	++	++ (medium)
bibliographies	++	+++	+++ (medium)
other			Front and Back Mattersection types, image legends, indexes
5) What are the purposes of the mark-up? One just cannot “tag everything”, as no single encoding of a text is going to be equally suitable for all thinkable purposes. Three main categories can be seen as:			
a) rendition/representation of the text in HTML, PDF, ePub, or other formats	+++	+++	+++
b) archiving of the text for long term preservation	++	+++	++
c) analysis, data mining, and other processing	+++	++	+++
6) What are the policies and practices for the handing of non-textual features such as illustrations, inserted plates, fold-out maps, etc.?			
a) how should multi-column text be handled?	Multi-column text normalized into single column	According to NLM publishing and archiving Tag Suite.	Currently most layout elements such as this are ignored unless columns numbered separately in original, in which case each column is treated as if it were a page.
b) what are the policies and practices regarding overlapping hierarchies in the text (say, a significant section starts in one chapter and concludes in another chapter of a book)?	Not encountered so far, so no respective policy – most documents marked in TaxonX are journals or journal articles, which next to never exhibit overlapping hierarchies	n/a in prospective publishing	+ treatments are dated from the first date of publication; supplements handled separately.
